# Predictive value of methylene blue combined with indocyanine green in sentinel lymph node metastasis in breast cancer: a prospective pilot cohort study

**DOI:** 10.3389/fonc.2024.1433907

**Published:** 2024-10-09

**Authors:** Zecheng He, Fan Guo, Yuhan Liu, Yan Lin, Changjun Wang, Yidong Zhou, Qiang Sun

**Affiliations:** ^1^ Department of Breast Surgery, Peking Union Medical College Hospital, Chinese Academy of Medical Sciences and Peking Union Medical College, Beijing, China; ^2^ Department of Plastic Surgery, Plastic Surgery Hospital, Chinese Academy of Medical Sciences and Peking Union Medical College, Beijing, China; ^3^ Department of Breast Surgery, Beijing Longfu Hospital, Beijing, China

**Keywords:** breast cancer, sentinel lymph node, metastasis, methylene blue, indocyanine green, risk prediction model

## Abstract

**Background:**

The status of sentinel lymph nodes is crucial for prognosis and treatment decisions in breast cancer patients. This study aimed to evaluate the predictive value of combined methylene blue and indocyanine green for sentinel lymph node metastasis in breast cancer.

**Methods:**

This prospective cohort study enrolled 90 clinically node-negative breast cancer patients. Methylene blue and indocyanine green were injected locally before surgery. Sentinel lymph nodes were grouped based on fluorescence intensity and methylene blue staining. A binary logistic regression model was established using 285 lymph node groups to predict metastatic risk.

**Results:**

A total of 475 lymph nodes were identified, with 33 being metastatic. The metastatic risk reached 70% for partially blue-stained and weakly fluorescent lymph nodes between 1-2 cm. The model revealed associations between lymph node size, dye staining patterns, and metastatic risks (P<0.05). The AUC of the ROC curve was 0.855.

**Conclusions:**

The staining pattern of combined methylene blue and indocyanine green could predict risks of sentinel lymph node metastasis and facilitate rapid intraoperative identification of high-risk lymph nodes.

## Highlights

**What is already known on this topic?**

- Sentinel lymph node biopsy (SLNB) using tracers like methylene blue (MB) and indocyanine green (ICG) is commonly used to assess axillary nodal status in clinically node-negative breast cancer patients.
- The relationship between MB and ICG staining patterns and sentinel lymph node (SLN) metastasis risk is not well established, with limited and inconclusive evidence from a few studies.
- It is unclear if the combined staining pattern of MB and ICG can reliably predict SLN metastatic status in breast cancer.

**What this study adds?**

- The study found that the staining pattern of combined MB and ICG has predictive value for breast cancer SLN metastasis.
- The metastatic risk could be as high as 70% for partially blue-stained and weakly fluorescent SLNs between 1-2 cm in size.
- A risk prediction model was developed showing associations between lymph node size, MB/ICG staining patterns, and SLN metastasis risk.
- Possible mechanisms are discussed for how tumor burden may impact lymphatic flow and tracer uptake, leading to the observed staining patterns in metastatic SLNs.

**How this study might affect research, practice or policy?**

- Based on the integrated indocyanine green (ICG) and methylene blue (MB) lymphatic mapping characteristics, the risk of sentinel lymph node (SLN) metastasis can be preliminarily predicted to screen out high-risk lymph nodes.
- Our study may provide a reference for intraoperative pathological diagnosis and facilitate communication between surgeons and pathologists, but it cannot completely replace frozen section examination.
- In the long run, with the improvement of predictive models, it may be possible to partially change the current clinical strategies and procedures for SLN management.

## 1 Introduction

Breast cancer is one of the most common malignancies globally, with approximately 2.2 million new cases and 680,000 related deaths in 2020, and its incidence and mortality rates continue rising (1). Early mammography and ultrasonography can detect breast cancer at earlier stages (2–4). Core needle biopsy helps confirm pathological diagnosis and guide treatment decisions. Compared to other solid tumors, breast cancer relies more heavily on lymphatic metastasis (5). Accurate evaluation of axillary lymph node involvement is equally critical for prognostication. Now sentinel lymph node biopsy (SLNB) is considered an accurate means to assess axillary nodal status in clinically node-negative breast cancer patients, avoiding unnecessary axillary dissection, reducing surgical morbidities like lymphedema, sensory and motor dysfunction, thereby improving quality of life (6).

Sentinel lymph node biopsy for breast cancer originated in the 1990s with the use of radioactive isotope tracers for lymph node localization (7). Common radioactive tracers during this period included 99mTc-labeled sulfur colloid (8). Currently, the commonly used tracers for sentinel lymph node biopsy in breast cancer include radioactive isotopes, blue dyes, and indocyanine green (ICG). Radioactive isotopes such as 99mTc-nanocolloid albumin have the advantage of high detection rates but require radioactive medicine equipment and specialized operators (8, 9). Blue dyes like methylene blue (MB) and isosulfan blue are more convenient and easy to use, but visual localization is less accurate compared to radioactive tracers (10, 11). Indocyanine green enables real-time fluorescence imaging without radiation exposure, but requires specialized imaging systems (12). And it is worth mentioning that ICG has been widely utilized in the identification and clearance of lymph nodes in colorectal surgery, providing us with a better understanding of many technical details related to ICG (13). In general, each tracer has its own unique strengths and limitations.

Recently, methylene blue and indocyanine green have been widely used in sentinel lymph node biopsy for breast cancer. Studies have shown that the combined use of methylene blue and indocyanine green can detect sentinel lymph nodes (SLN) missed by either tracer alone, reducing false negative rates and accurately determining nodal status (14–16). A few studies have explored the relationship between methylene blue and indocyanine green staining and sentinel lymph node metastasis risk: J.R. van der Vorst et al. found that the risk of metastasis increased in lymph nodes with absent or decreased indocyanine green staining (17), but Tagaya et al.’s study showed no clear association between indocyanine green staining and lymph node metastasis (18). Regarding methylene blue, Wishart et al.’s study demonstrated that methylene blue staining could not predict lymph node metastasis (15). However, current evidence is insufficient to conclusively determine if methylene blue and indocyanine green staining can reliably predict sentinel lymph node metastatic status.

In this study, we utilized data from a cohort of breast cancer patients who underwent sentinel lymph node biopsy to comprehensively analyze the predictive value of combined methylene blue and indocyanine green for breast cancer sentinel lymph node metastasis. We exploratorily established a multivariate risk prediction model containing only sentinel lymph node-related characteristics, providing new insights for further developing a more objective, comprehensive, and clinically translatable risk prediction model.

## 2 Methods

### 2.1 Study design

This our prospective cohort study was conducted at Beijing Longfu Hospital from March 1, 2022, to February 28, 2023. Ethical approval from the Ethical Committee of Beijing Longfu Hospital with certificate number of LFYYLL-2022-08, and informed consent of the patients were obtained. All patients signed the consent forms and expressed agreement to participate in this study. The inclusion criteria were as follows: (1) age over 18 years; (2) initial diagnosis of breast cancer confirmed by either a core needle biopsy or an excisional biopsy; (3) absence of suspicious enlarged lymph nodes on axillary palpation and ultrasonography; (4) consent to undergo sentinel lymph node biopsy surgery; (5) agreement to the use of methylene blue and indocyanine green for dual-tracer mapping. The exclusion criteria included: (1) male breast cancer; (2) pregnancy; (3) bilateral breast cancer; (4) previous neoadjuvant therapy; (5) prior axillary surgery on the affected side; (6) allergy to methylene blue or indocyanine green. We designated the acquisition of complete paraffin pathology reports for all sentinel lymph nodes as the primary endpoint. Furthermore, this study only included a short-term postoperative follow-up at one month to assess the occurrence of adverse events such as lymphedema, sensory or motor deficits, skin staining, or skin necrosis.

### 2.2 Tracers selection and preparation

In this study, the preparation method for ICG (Ruidu^®^, Dandongyichuang Pharmaceutical Co., LTD., Liaoning, China, 25mg) is as follows: 5.0 ml sterile water is drawn and mixed with the ICG powder to create 5.0 mg/ml stock solution. Then, 1.25 ml of the stock solution is combined with 5.0 ml sterile water to produce 1.0 mg/ml ICG diluent. The methylene blue (Methylthioninium Chloride Injection, JUMPCAN Pharmaceutical Group Co., LTD., Jiangsu, China, 10mg/ml) used in this study is ready for injection without the need for prior preparation.

### 2.3 Surgical procedures and intraoperative examination

Preoperatively, 0.4 mL of MB (10 mg/ml) and 0.4 mL of ICG diluent (1.0 mg/ml) were injected intradermally around the areola on the affected side, and we usually choose two normal areas of skin and inject both tracers evenly. The patient was then instructed to gently massage the breast circularly at the injection site for 10 minutes. Subsequently, the patient entered the operating room and took the surgical position. After turning off the surgical lights, real-time ICG lymphatic drainage was observed using the SES-CII multispectral fluorescence imaging system (Saiensi Technology Development Co., LTD, Shanxi, China). Fluorescence imaging of lymphatic tracts was traced from the areola toward the axilla to determine the location for SLNB, selecting the site where fluorescent signal faded in the axilla. If ICG fluorescence imaging failed, the conventional empirical axillary skin crease incision was made. Next, routine surgical area disinfection and sterile draping were performed, followed by local infiltration anesthesia. The axilla skin, subcutaneous tissue, and fascia was incised according to anatomical planes, resecting lymph nodes stained blue by methylene blue and fluorescent lymph nodes visualized by ICG fluorescence imaging carefully. Suspicious malignant palpable lymph nodes were also removed. If MB staining failed, all lymph nodes along the intercostobrachial nerve and lateral thoracic vein were removed conventionally (19). At least one sentinel lymph node is removed in each patient. When SLNs are removed during operation, the surrounding adipose tissue should be removed as thoroughly as possible to avoid interfering with the observation and judgment of MB staining and ICG fluorescence. Then, based on fluorescence intensity displayed by the imaging system, the harvested SLNs and surrounding adipose tissue were sorted into multiple groups, with only one lymph node per group if possible. Fluorescence intensity of each SLN group was photographed and recorded. MB staining status were documented for each group. Finally, all specimens were sent to the same experienced and skilled pathology team for frozen section, ensuring that each pathologist was completely blinded to fluorescence status when making diagnoses. The pathologist categorized the metastatic status of SLNs into positive or negative, and reported it to the surgeon during surgery. According to the pathological results of SLNB, axillary lymph node dissection (ALND) should be performed in patients with any of the following conditions: (1) SLNB detected at least 3 metastatic SLNs; (2) SLNB detected 1-2 metastatic SLN, but patients are unwilling to undergo postoperative radiotherapy (6, 20). In addition, the surgeon should consider the patient’s informed consent before surgery and physical condition when deciding whether to perform ALND.

In this study, we use the online image processing service IMAGE COLOR SUMMARIZER (https://mk.bcgsc.ca/color-summarizer/?analyze=), and every fluorescent SLN is summarized by dividing into five color distributions based on the number of pixels, and the percentage of each color is calculated. Then depending on the size of the fluorescence intensity ratio (FIR) for each SLN group, ICG staining status were recorded as *‘strongly positive’ (FIR≥1.00)*, *‘weakly positive’ (0<FIR<1.00)*, or *‘negative’ (FIR=0)*. Please refer to **Supplementary Material S1** for the FIR calculation method and process designed by us. In addition, MB staining status were recorded as *‘totally stained’*, *‘partially stained’*, or *‘negative’* based on cautious judgment by the surgeon, first assistant, and pathologist back-to-back. In detail, sentinel lymph nodes (SLNs) were categorized as totally or partially stained according to the range of blue dye within the SLN itself. If only the surrounding afferent lymphatic vessels were stained blue while the SLN itself was not, the SLN was judged as partially stained.

### 2.4 Pathological analysis

Postoperatively, all the excised SLNs and remaining axillary lymph nodes from each patient were subjected to formalin-fixed and paraffin-embedded tissue sections by the same group of pathologists, in accordance with the 8th edition American Joint Committee on Cancer (AJCC) Cancer Staging Manual. Immunohistochemistry was performed for the confirmation of suspected metastases. Final pathology reports were issued individually according to the number of specimen groups submitted intraoperatively.

### 2.5 Data collection

Four types of data were collected in this study. First, patient demographic information and histopathological characteristics of the primary breast tumors were retrieved from medical records. Second, the number of resected SLNs, staining status of each SLN group, maximum diameter (MD), and metastatic status of each SLN group on final pathology were documented for each patient. Third, fluorescence imaging system was utilized to delineate regions of interest (ROI) around each SLN group and quantify mean and maximum fluorescence values within these regions for all patients. Finally, postoperative adverse events occurring within one month were tracked through the hospital follow-up system for each patient.

### 2.6 Statistical analysis

Continuous variables were presented as mean ± standard deviation or median (interquartile range) based on normality assessed by the Kolmogorov-Smirnov test. Categorical variables were expressed as frequency (percentage). Patients and sentinel lymph nodes were categorized into metastasis positive or negative groups. Categorical variables were compared between groups using chi-square test or Fisher’s exact test or Chi-square goodness of fit test. Continuous variables were compared by independent samples t-test or Wilcoxon rank-sum test. Sentinel lymph nodes were further divided into metastatic positive or negative groups. Univariable and multivariable binary logistic regression analyses were performed to examine the association between lymph node characteristics (number of SLNs per group, size of fluorescent ROI, fluorescence values, node diameter, staining status) and metastasis risk. A prediction model was developed and receiver operating characteristic (ROC) curve analysis was conducted. Statistical significance was defined as two-sided p<0.05. Analyses were performed using SPSS software (version 29.0.1, IBM Corp., Armonk, NY, USA).

## 3 Results

### 3.1 Patient characteristics

Based on the aforementioned criteria, a total of 90 breast cancer patients were enrolled consecutively in the study. The patients were mainly middle-aged women between 40 and 60 years old, accounting for 68.9%. And the proportion of normal-weight patients was 52.2%, which was basically the same as that of underweight, overweight and obese patients combined. 55.6% of the patients were premenopausal, and 52.2% of the patients had primary lesions in the left breast. In terms of the history of breast tumor operation, most of the patients had never received surgical intervention, accounting for 64.4%; a small number of patients only underwent fine needle biopsy, accounting for 24.4%; only 10 patients had previously undergone tumor excision biopsy or mymerton rotary incision therapy, accounting for 11.1%. All enrolled patients underwent SLNB according to the pathological results after resection of breast tumors. And it is important that a total of 21 patients developed SLN metastasis, of which 19 patients received further ALND according to ASCO guidelines and updated criteria, and 2 patients did not receive ALND because they had fewer than 3 SLN metastases and were willing to undergo axillary radiotherapy. For detailed information on patient characteristics, please refer to **Table 1**, which also contains specific types of breast surgery and their respective percentages, as well as information on postoperative pathology of breast tumors.

**Table 1:** Baseline characteristics of enrolled patients.

Characteristics	Patients, n	Value (%)
**Total**	90	
Age (years)		
<40	15	16.7
40-60	62	68.9
>60	13	14.4
BMI (kg/m2)		
≤ 18.4	8	8.9
18.5–23.9	47	52.2
24.0–27.9	25	27.8
≥28.0	10	11.1
Menopause		
Pre	50	55.6
Post	40	44.4
Laterality		
Left	47	52.2
Right	43	47.8
History of breast tumor operation		
Unoperated	58	64.4
Needle biopsy	22	24.4
Excisional biopsy	8	8.9
Mymerton rotary incision therapy	2	2.2
Type of breast surgery		
Excisional biopsy + Breast-conserving surgery	33	36.7
Excisional biopsy + Mastectomy	23	25.6
Breast-conserving surgery	12	13.3
Mastectomy	20	22.2
Mastectomy + Reconstruction surgery	2	2.2
Type of axillary surgery		
SLNB	90	100.0
ALND	19	21.1
SLN metastasis status		
Positive	21	23.3
Negative	69	76.7
Histological type		
Invasive ductal carcinoma	77	85.6
Others	13	14.4
pT stage		
pTis	7	7.8
pT1	44	48.9
pT2	33	36.7
Unknown	6	6.7
Molecular subtype		
Luminal A	8	8.9
Luminal B	51	56.6
Her-2 enriched	5	5.6
Triple negative	9	10.0
Unknown	17	18.9

### 3.2 Comparisons between sentinel lymph node positive and negative groups

**Figure 1:**
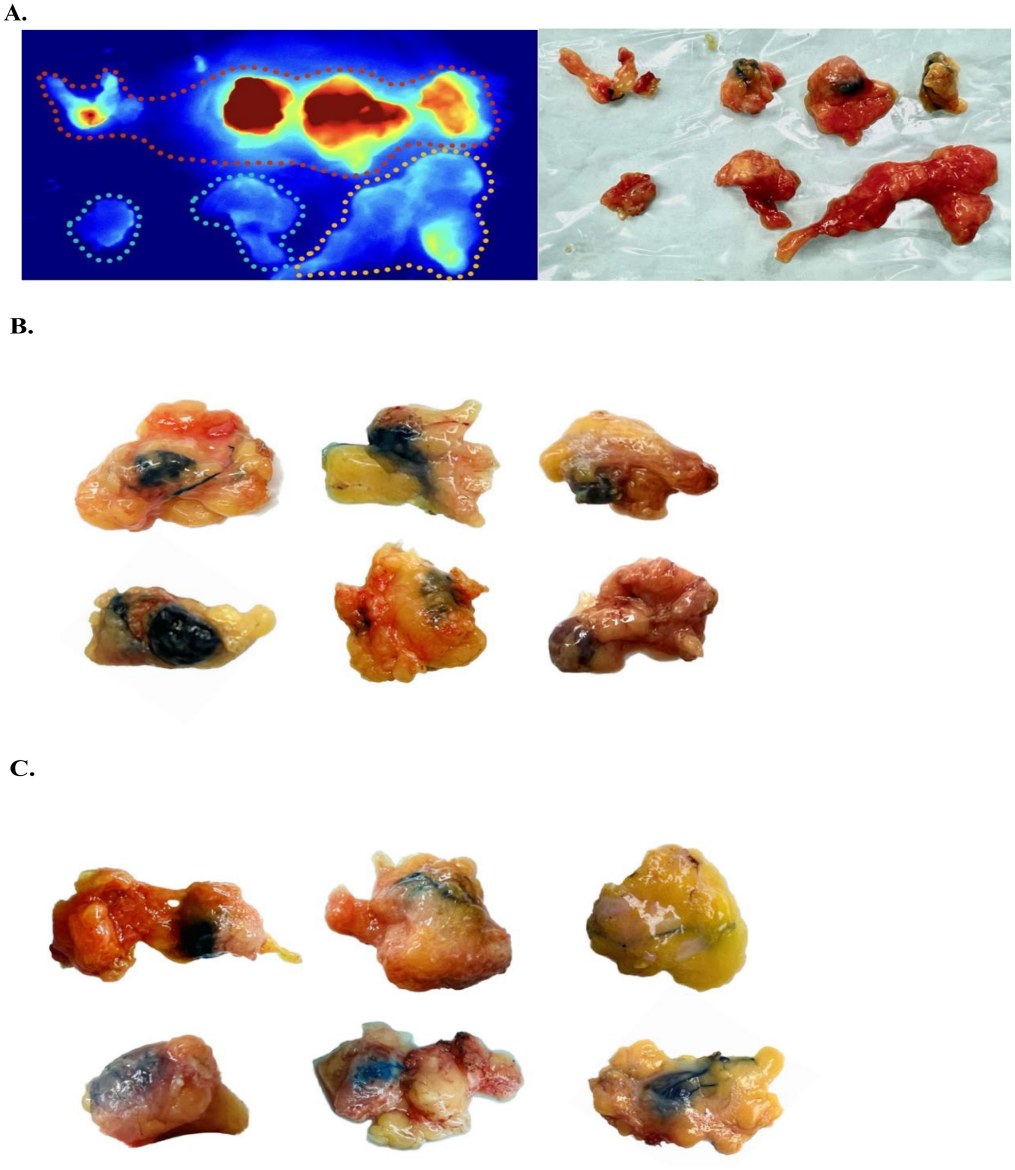
Sentinel lymph node grouping and staining characteristics. **(A)** Sentinel lymph nodes exhibiting fluorescence intensity within the red, yellow, and blue dashed boundaries are recorded as *‘Strongly positive’*, *‘Weakly positive’* and *‘Negative’*, respectively. **(B)** Macroscopic observation of several typical *‘Totally stained’* sentinel lymph nodes. Intraoperatively, these sentinel lymph nodes (SLNs) were observed to exhibit an overall blue coloration. **(C)** Gross observation of sentinel lymph nodes with partial blue staining or blue staining of perilymphatic tracts only, all recorded as *‘Partially Stained’*.

According to ICG staining status of the resected SLNs of each patient, that is, the intensity of fluorescence imaging, each sample was divided into several groups, and frozen section was performed on each group of lymph nodes, while MB staining status of each group of lymph nodes was recorded. **Figure 1** and **Supplementary Material S1** show an overview of the grouping of SLNs using the fluorescence imager and exhibits MB staining status of some lymph nodes.

**Figure 2:**
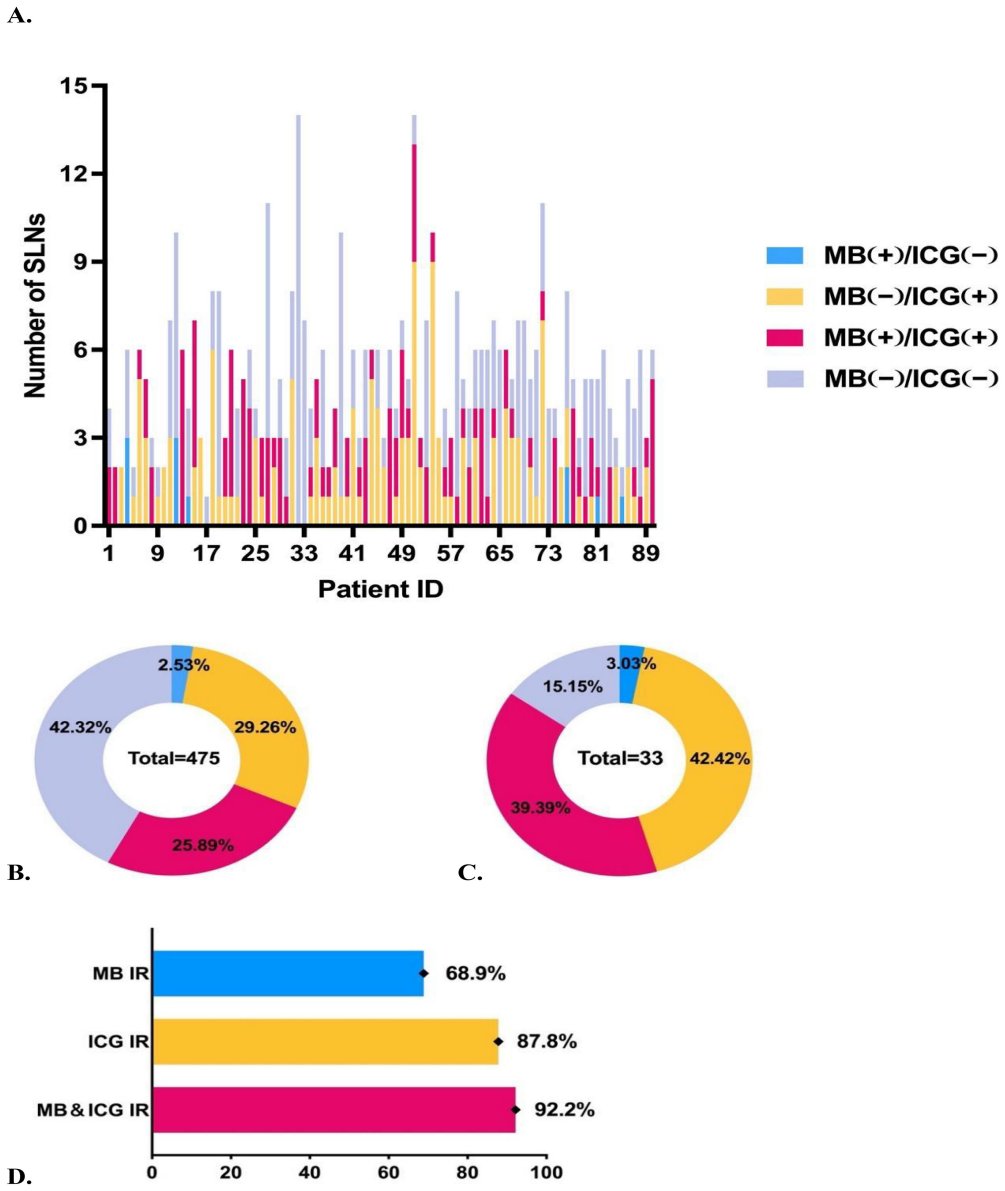
Overview of staining status of sentinel lymph nodes. **(A)** Number of sentinel lymph nodes excised and staining status for each patient. **(B)** Staining status and proportions of all sentinel lymph nodes. **(C)** Staining status and proportions of metastatic sentinel lymph nodes. **(D)** Comparison of identification rates using different tracers.

After excluding 20 groups with ICG staining of adipose tissue, we analyzed 285 sets of SLN samples comprising a total of 475 lymph nodes. Of these, 33 lymph nodes were confirmed to have metastatic involvement, with 5.3 ± 2.5 lymph nodes detected per patient (**Figures 2A–C**). And the identification rates (IR) of MB, ICG, and the combined MB and ICG dual-tracer method were 68.9, 87.8%, and 92.2%, respectively (**Figure 2D**). The false-negative rate of frozen section was 12.1%, and the concordance rate between frozen section and paraffin-embedded pathology was 96.7%.

Then, based on the presence or absence of metastasis in the SLNs, the 90 patients were divided into SLN (+) and SLN (−) groups (**Table 2**). We analyzed baseline characteristics such as age, menopause, and body mass index (BMI) between the two groups and found no significant differences. However, we observed that staining status of SLNs differed significantly between groups (p=0.005), with more SLN positive groups exhibiting partial MB staining.

**Table 2:** Patient group comparison based on metastatic status of sentinel lymph nodes.

	SLN(−)	SLN(+)	χ^2^	P value
**Number of patients, n (%)**	69(76.7%)	21(23.3%)		
**Age (years)**			2.982	0.225
<40	9(13.0)	6(28.6)		
40-60	49(71.0)	13(61.9)		
>60	11(16.0)	2(9.5)		
**BMI (kg/m2)^@^**			−	0.261
≤ 18.4	4(5.6)	4(19.0)		
18.5–23.9	38(55.1)	9(42.9)		
24.0–27.9	20(30.0)	5(23.8)		
≥28.0	7(9.3)	3(14.3)		
**Menopause**			1.370	0.242
Pre	36(52.2)	14(66.7)		
Post	33(47.8)	7(33.3)		
**Laterality**			0.000	0.987
Left	36(52.2)	11(52.3)		
Right	33(47.8)	10(47.7)		
**Operation History^#^**			0.000	1.000
Resection	8(11.6)	2(9.5)		
Others	61(88.4)	19(90.5)		
**Number of SLNs, n (%)**	442(93.1%)	33(6.9%)		
**SLN staining status^@^**			−	0.005*
MB(+)/ICG(−)	11(2.5)	1(3.0)		
MB(−)/ICG(+)	125(28.3)	14(42.4)		
MB(+)/ICG(+)	110(24.9)	13(39.4)		
MB(−)/ICG(−)	196(44.3)	5(15.2)		

*P<0.05.

@Fisher’s exact test.

#Chi-square goodness of fit test.

### 3.3 Correlations of sentinel lymph node characteristics and metastatic status

Similarly, based on intragroup detection of metastasis in SLNs, we divided the 285 sets of lymph nodes submitted into SLN (+) and SLN (−) group. An analysis of factors specific to SLNs was conducted. We observed differences between SLN (+) and SLN (−) groups in terms of ROI, fluorescence maximum value (FMV), maximum lymph node diameter, and staining status, with P-values all being less than 0.05 (**Table 3**).

**Table 3:** Submitted sentinel lymph node group comparison based on metastatic status of sentinel lymph nodes.

	Total *(n = 285)*	SLN(−) *(n = 257)*	SLN(+) *(n = 28)*	Statistic	P value
**Number of SLNs** *Mean ± SD*	1.65 ± 1.05	1.64 ± 1.05	1.71 ± 1.08	t=-0.364	0.716
**ROI** *Mean ± SD*	4.59 ± 3.73	4.43 ± 3.72	6.09 ± 3.60	t=-2.242	0.026*
**FAV** *Mean ± SD*	78.19 ± 63.79	76.25 ± 63.08	96.04 ± 68.63	t=-1.563	0.119
**FMV** *n (%)*				χ^2^ = 8.786	0.012*
<130	111 (38.95)	107 (41.63)	4 (14.29)		
130~200	40 (14.04)	36 (14.01)	4 (14.29)		
>200	134 (47.02)	114 (44.36)	20 (71.43)		
**MD of SLNs** *n (%)*				−	0.017*
<1.0cm	181 (63.51)	170 (66.15)	11 (39.29)		
1.0~2.0cm	101 (35.44)	84 (32.68)	17 (60.71)		
>2.0cm	3 (1.05)	3 (1.17)	0 (0.00)		
**MB staining status** *n (%)*				χ^2^ =64.787	<.001*
Negative	198 (69.47)	182 (70.82)	16 (57.14)		
Partially stained	14 (4.91)	4 (1.56)	10 (35.71)		
Totally stained	73 (25.61)	71 (27.63)	2 (7.14)		
**ICG staining status** *n (%)*				χ^2^ = 10.380	0.006*
Negative	122 (42.81)	118 (45.91)	4 (14.29)		
Weakly positive	38 (13.33)	32 (12.45)	6 (21.43)		
Strongly positive	125 (43.86)	107 (41.63)	18 (64.29)		

*P<0.05.

ROI, region of interest; FAV, fluorescence average value; FMV, fluorescence maximum value; MD, maximum diameter.

**Figure 3:**
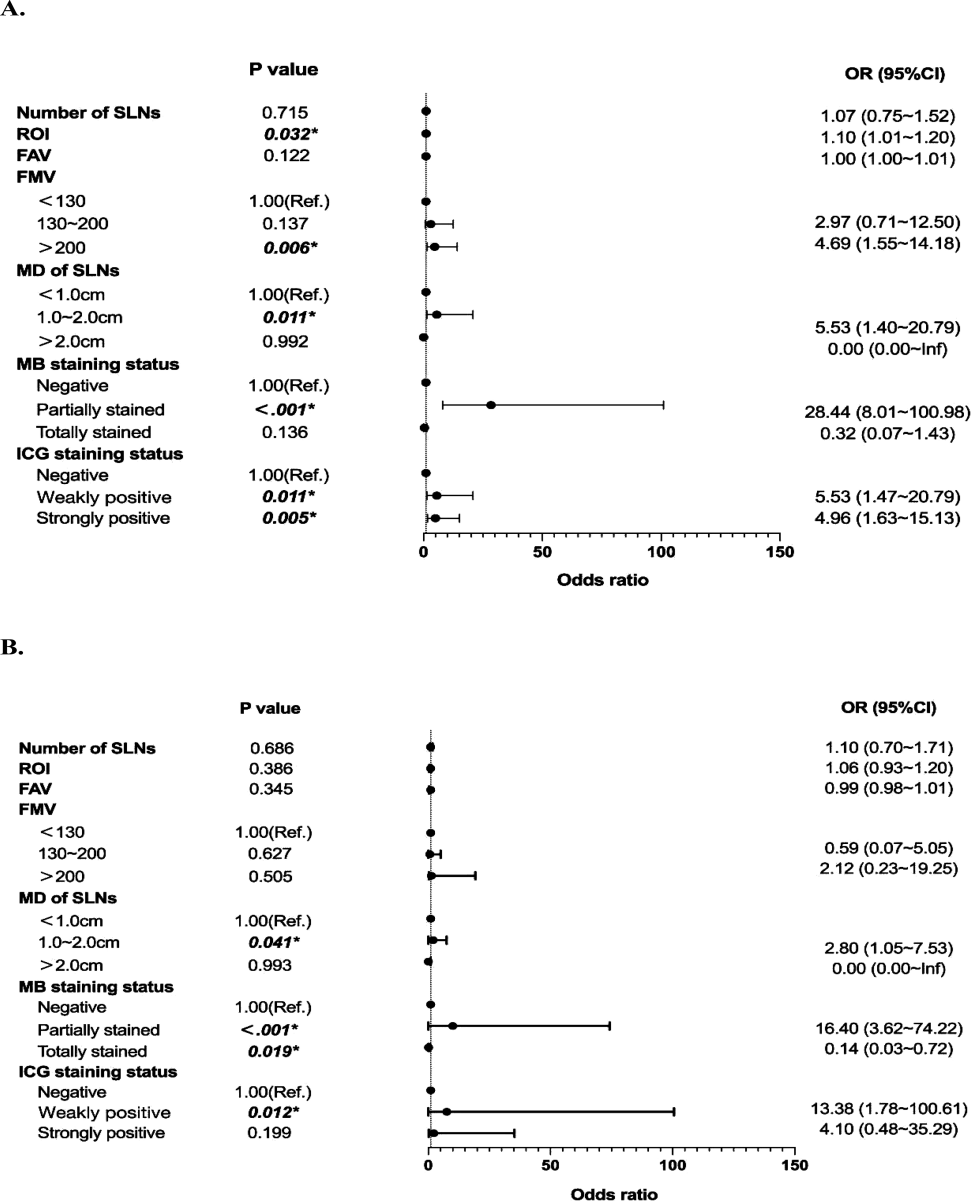
Univariate and multivariate analyses based on sentinel lymph node characteristics. **(A)** Forest Plot of Univariate Binary Logistic Regression Analysis. **(B)** Forest Plot of Multivariate Binary Logistic Regression Analysis.

**Figure 4:**
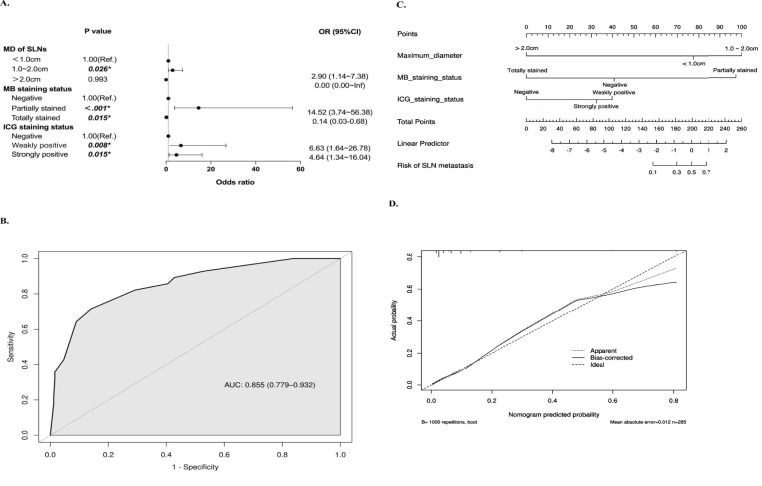
Development of the sentinel lymph node metastasis risk prediction model. **(A)** Forest Plot of Multivariate Analysis for Metastasis Risk Factors. **(B)** ROC Curve and AUC (95%CI) Value of the Predictive Model based on the maximum diameter of sentinel lymph nodes, methylene blue and indocyanine green staining status. **(C)** Nomogram of the Predictive Model with Corresponding Points for Metastasis Risk Factors. **(D)** Calibration Curve of the Nomogram for Prediction.

We further performed univariate and multivariate binary logistic regression analyses to explore the association between SLN characteristics and metastasis. Compared to SLNs less than 1 cm, those between 1-2 cm were associated with higher metastatic risk (OR=2.90, 95%CI=1.14~7.38, P=0.026). ‘*Partially stained*’ SLNs, versus MB unstained, also conferred greater metastatic risk (OR=16.40, 95%CI=3.62~74.22, P<0.001). Similarly, ‘*Weakly positive*’ SLNs were linked to higher probability of metastasis compared to lymph nodes with negative fluorescence (OR=13.38, 95%CI=1.78~100.61, P=0.012) (**Figure 3**). Then, a model to predict metastatic risk in SLNs was constructed using maximum sentinel lymph node diameter, MB and ICG staining status as predictors (**Figure 4A**). The model demonstrated that SLNs between 1-2 cm conferred higher metastatic risk compared to those <1 cm (OR=2.90, 95%CI=1.14~7.38, P=0.026). Partially methylene blue stained SLNs carried greater metastatic risk versus lymph nodes with negative staining (OR=14.52, 95%CI=3.74~56.38, P<0.001). Both strongly and weakly indocyanine green fluorescent SLNs were associated with higher probability of metastasis compared to lymph nodes with no fluorescence (‘*Strongly positive*’: OR=4.64, 95%CI=1.34~16.04, P=0.015; ‘*Weakly positive*’: OR=6.63, 95%CI=1.64~26.78, P=0.008). And the Hosmer-Lemeshow test yielded a significance level of 0.96, indicating that the model demonstrates good fit. Based on the above results, the receiver operating characteristic curve was generated and plotted for the model (AUC=0.855, 95%CI=0.779~0.932) (**Figure 4B**). The SLN metastasis risk prediction model was visualized using a nomogram, and the calibration curve of the nomogram fitted well (**Figures 4C, D**).

### 3.4 Short-term postoperative complications

During our short-term 1-month postoperative follow-up in this study, we found that none of the 71 patients who underwent SLNB alone developed complications such as lymphedema, sensory or motor dysfunction, skin staining or necrosis at the injection site. However, the 19 patients who underwent ALND all exhibited varying degrees of sensory abnormalities in the axilla and mild restricted mobility of the ipsilateral upper limb.

## 4 Discussion

A previous study has found that in SLNB using the dual-tracer method (combined MB and ICG) for breast cancer, all 19 metastatic SLNs were fluorescent positive, but up to 8 lymph nodes were not stained by MB (21). Another similar study has also reported 2 cases where only fluorescent SLNs were found to be metastatic (22). However, the extremely limited relevant data available has not been able to conclusively correlate the staining patterns of MB and ICG with metastatic status in SLNs.

Our study found that the staining pattern of combined MB and ICG has a certain predictive value for breast cancer SLN metastasis: The metastatic risk could be as high as 70% for partially blue-stained and weakly fluorescent SLNs with the maximum diameter between 1-2 cm. There are three possible reasons for this phenomenon: First, studies have shown that macrometastases in SLNs increase nodal volume and pressure, obstructing lymph flow and causing tracers to drain to non-sentinel lymph nodes without tumor deposits (23–25). Specifically, crowded tumor cells secrete cytokines inducing cytoskeletal stiffness, endothelial proliferation, and immune cell hyperplasia, all limiting dye accumulation (26–30). Micrometastases do not significantly impact volume or pressure (31), explaining our finding of strong dual staining in the single isolated tumor cell metastasis. Second, prolonged tumor cell invasion may reduce antigen-induced lymphocytes and impair phagocytic uptake of tracers by macrophages (32). Third, some studies show tumor burden does not restrict fluorescent flow, possibly because smaller indocyanine green molecules penetrate easier than blue dyes (33). Although ICG and MB can assist us in finding the approximate location of SLNs, true pathologic SLNs may be missed during surgery, given the fact that metastatic LNs may not be able to absorb the tracer as much. Therefore, we suggest that more attention should be paid to SLNs that are fluorescent but not blue stained, as they are likely to have metastasis. Removing these suspected LNs improves diagnostic accuracy and specificity, explaining the dominance of metastatic nodes with absent blue staining.

In addition, among the 257 groups of non-metastasis SLNs in our study, 6 groups were ICG and MB negative. However, in the 28 groups of metastatic SLNs, only 1 group was ICG negative but showed partial MB staining. We speculate the lymph nodes with dual negative staining may represent two categories: first, the fake SLNs without tracer uptake as described before; second, the true sentinel nodes with possible macrometastases. Studies have shown up to 36.4% of ICG negative lymph nodes contained macrometastases, as extensive tumor obstructions restrict dye penetration (34). The single group of partial MB staining and ICG negative could be attributed to quenching or improper injection preventing imaging. A nonnegligible research indicates high indocyanine green concentrations actually form fluorescing aggregates that evade detection, with high doses causing self-quenching and failed visualization (35).

Overall, during SLNB, high-risk SLNs could be preselected for intraoperative frozen section based on our study findings, which may shorten the turnaround time of frozen section results, reduce operative time, and improve work efficiency.

Several limitations of our study should be considered. Firstly, the sample size was small with only 90 patients, and all patients were from a single center, thus the representativeness of the results was limited. Secondly, we found that the maximum fluorescence value of each group of SLNs calculated by the fluorescence imager under the standard measurement distance and angle did not entirely match the color of the image presented in real time. For example, some SLNs with the maximum fluorescence value of 0 showed red in the image, but often such SLNs that appear red have FIR greater than 1.00 and are classified as *‘strongly positive’*. Therefore, the classification of ICG fluorescence intensity of SLN in this study is still subjective, although semi-quantitative analysis is carried out by calculating FIR. Future studies may try to develop standardized and quantitative fluorescence intensity classification methods to improve the objectivity and repeatability of results. Thirdly, although the surrounding adipose tissue was removed as much as possible when SLNs were removed and divided into groups during the operation, it was inevitable that a small amount of adipose tissue remained in each group of SLNs in this study, which may have a certain impact on the interpretation of the results and is an aspect that needs to be improved. In addition, the influence factors such as ICG injection dose and excitation light intensity should be controlled to improve the consistency of classification. Fourth, validation using a verification cohort or independent validation dataset was lacking, thus the robustness of the model results was questionable.

Moving forward, we should attempt to further expand the sample size with a multicenter study design, establish more standardized criteria, and develop risk prediction models incorporating various factors through more sophisticated machine learning and deep learning approaches.

## 5 Conclusions

The staining pattern of combined methylene blue and indocyanine green could predict risks of sentinel lymph node metastasis and facilitate rapid intraoperative identification of high-risk lymph nodes. However, we need to stress that frozen section examination remains the cornerstone for detecting SLN metastatic risk. More studies are still needed to better understand the technical details of tracer staining before it can be established as a reliable means of SLN metastatic risk detection on its own. Whether as an adjunct to frozen section analysis or not, utilizing tracer staining for risk detection requires more technological advancements, especially in the quantitative analysis of ICG fluorescence.

## Data Availability

The raw data supporting the conclusions of this article will be made available by the authors, without undue reservation.
