# Supplementary material for: Predictive value of methylene blue combined with indocyanine green in sentinel lymph node metastasis in breast cancer: a prospective pilot cohort study
Source: Front Oncol. 2024 Oct 9;14:1433907. doi: 10.3389/fonc.2024.1433907 (PMC11496266; doi:10.3389/fonc.2024.1433907)
Supplement: Supplementary file 1 [file DataSheet1.pdf]

Supplement 1S.A

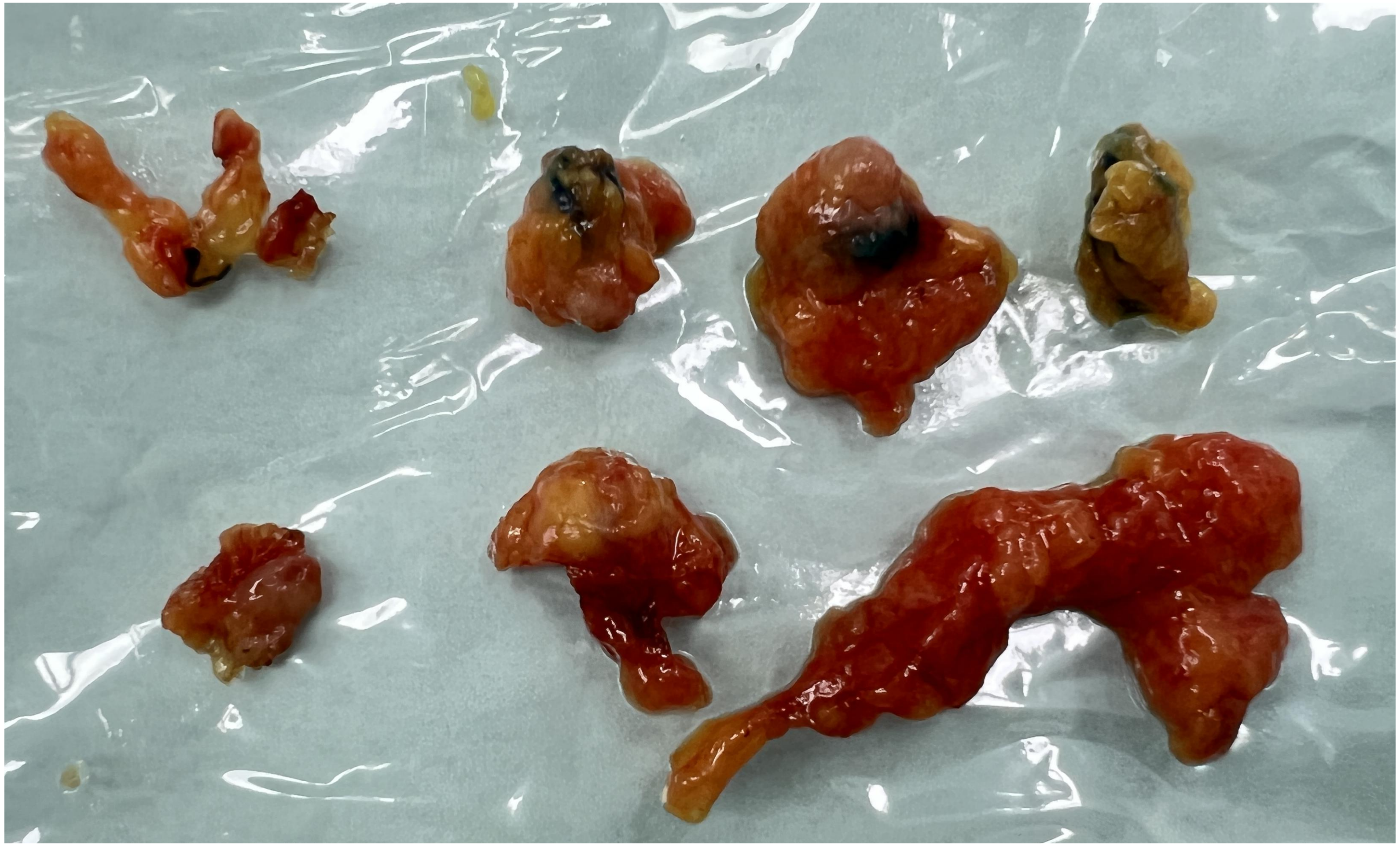

Supplement 1S.B

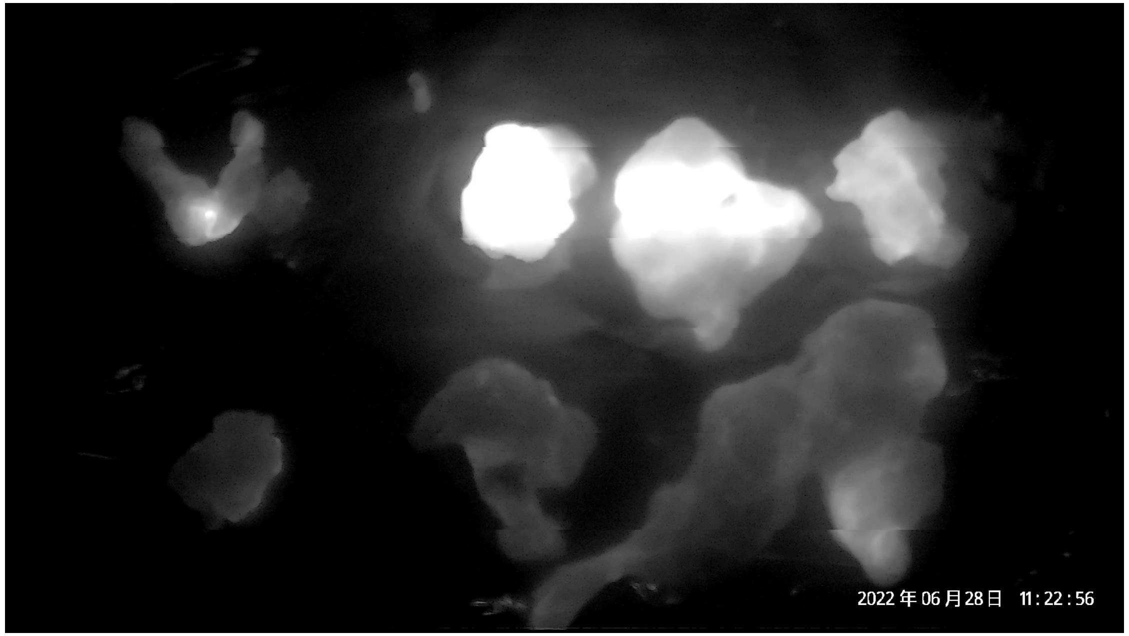

Supplement 1S.C

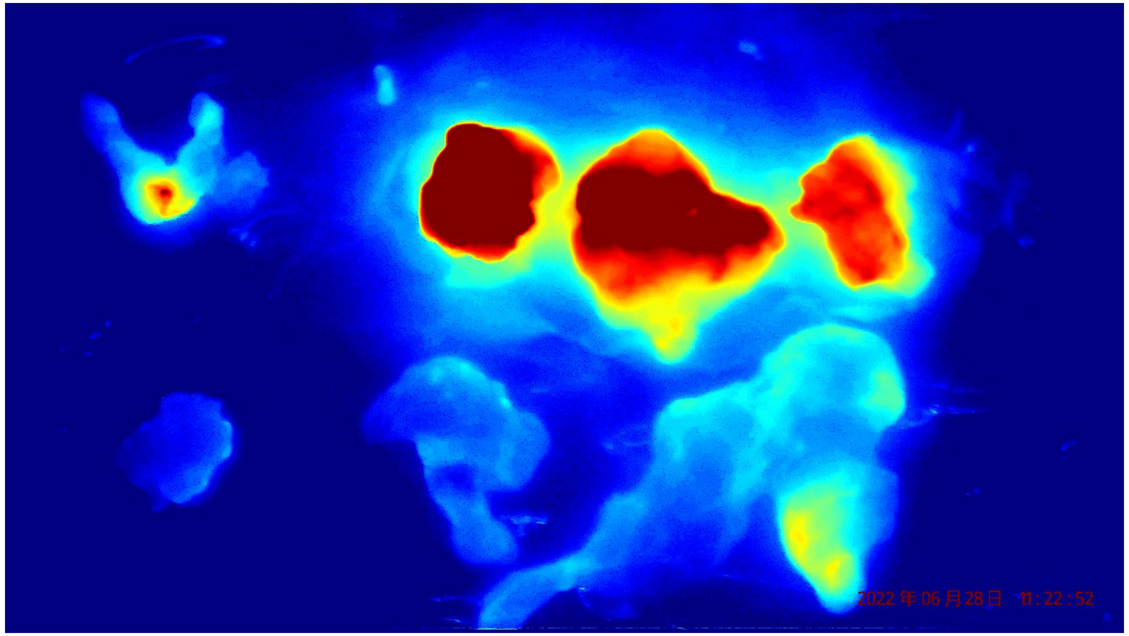

Supplement 1S.D

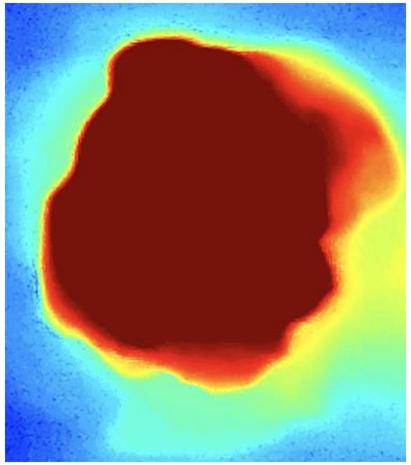

Fluorescence intensity ratio (FIR)

=(46.83%+12.66%)/15.07%

=3.95

COLOR CLUSTERS

Colors in the image were clustered into **5** groups (k-means). The average color of the colors for each cluster is shown. The name is the closest named color and its distance is shown using ΔE. The tags are the set of words formed by all named neighbours within ΔE ⩽ 5. The list of words above is the set of all unique words in this set of words.

Cluster colors, sized by number of pixels:

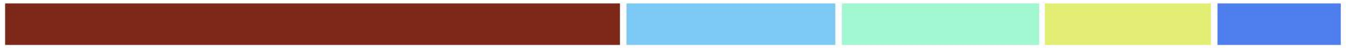

cluster	pixels	name	HEX	RGB	HSV	LCH	Lab	tags
	46.83%	146,47,29<br>countdown ΔE=4.8	#891F11	137 31 17	7 88 54	30 56 39	30 44 35	countdown scarlet
	15.99%	101,216,255<br>light brilliant cerulean ΔE=5.5	#5FCCFA	95 204 250	198 62 98	78 37 240	78 -18 -32	light brilliant cerulean
	15.07%	127,255,212<br>aquamarine ΔE=2.7	#84FBCE	132 251 206	157 47 98	91 45 166	91 -44 11	light aquamarine turquoise
	12.66%	213,231,81<br>brilliant apple green ΔE=3.5	#E0EE5D	224 238 93	66 61 94	91 71 109	91 -24 67	brilliant apple green
	9.45%	62,130,252<br>dodger blue ΔE=2.8	#3D81F6	61 129 246	218 75 97	55 68 286	55 19 -65	blueberry dodger blue

IMAGE CLUSTER PARTITIONS

Pixels of the image assigned to each cluster. The border is the color of the cluster as calculated by the average value of its pixels.

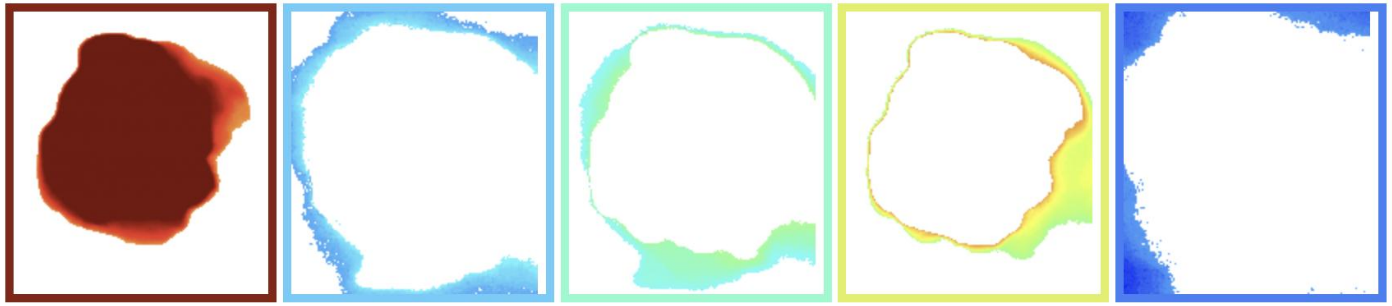

Supplement 1S.E

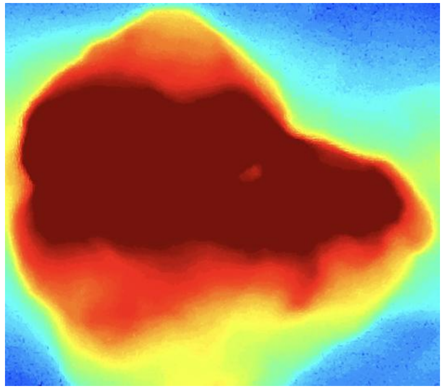

Fluorescence intensity ratio (FIR)

=(31.55%+20.76% +18.16)/13.78%

=5.11

COLOR CLUSTERS

Colors in the image were clustered into **5** groups (k-means). The average color of the colors for each cluster is shown. The name is the closest named color and its distance is shown using ΔE. The tags are the set of words formed by all named neighbours within ΔE ⩽ 5. The list of words above is the set of all unique words in this set of words.

Cluster colors, sized by number of pixels:

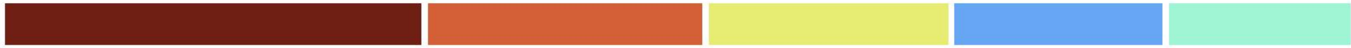

cluster	pixels	name	HEX	RGB	HSV	LCH	Lab	tags
	31.55%	119,15,5 burgundy ΔE=2.1	#7A150D	122 21 13	4 89 48	26 53 38	26 42 32	dark burgundy japanese jazz maple maroon oxide reddy up brown red
	20.76%	226,88,34 flame ΔE=2.8	#E55727	229 87 39	15 83 90	56 76 46	56 53 54	flame
	18.16%	226,230,77 canary ΔE=3.8	#E4ED5A	228 237 90	64 62 93	91 71 107	91 -21 68	brilliant apple canary green yellow
	15.75%	101,178,255 light brilliant azure ΔE=4.9	#50A8F8	80 168 248	208 68 97	67 47 268	67 -2 -47	light brilliant azure
	13.78%	126,244,204 light turquoise ΔE=2.1	#82F8D3	130 248 211	161 47 97	90 42 170	90 -42 7	light aqua aquamarine turquoise

IMAGE CLUSTER PARTITIONS

Pixels of the image assigned to each cluster. The border is the color of the cluster as calculated by the average value of its pixels.

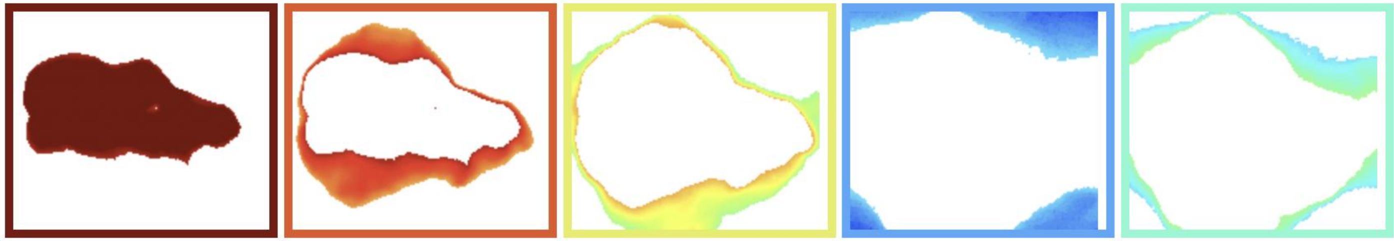

Supplement 1S.F

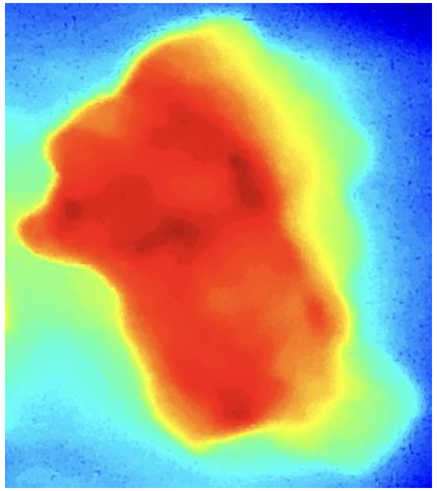

Fluorescence intensity ratio (FIR)

=(34.46%+18.82%)/17.87%

=2.98

COLOR CLUSTERS

Colors in the image were clustered into **5** groups (k-means). The average color of the colors for each cluster is shown. The name is the closest named color and its distance is shown using ΔE. The tags are the set of words formed by all named neighbours within ΔE ⩽ 5. The list of words above is the set of all unique words in this set of words.

Cluster colors, sized by number of pixels:

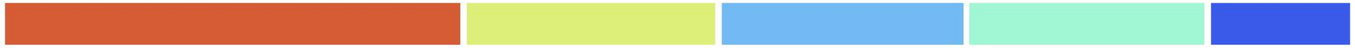

cluster	pixels	name	HEX	RGB	HSV	LCH	Lab	tags
	34.46%	242,85,42 flamingo ΔE=3.5	#E75126	231 81 38	13 83 91	55 78 44	55 56 54	flamingo
	18.82%	205,230,93 sublime ΔE=4.3	#D9F063	217 240 99	70 59 94	91 69 113	91 -26 64	sublime
	18.27%	101,197,255 light brilliant cornflower blue ΔE=3.8	#58BCF9	88 188 249	203 65 98	73 40 254	73 -11 -39	light brilliant cornflower maya blue
	17.87%	126,244,204 light turquoise ΔE=1.7	#83FAD1	131 250 209	160 47 98	91 44 168	91 -43 9	light aqua aquamarine turquoise
	10.58%	0,96,255 luminous vivid cobalt blue ΔE=4.5	#2F5BF2	47 91 242	226 81 95	45 89 296	45 39 -80	luminous vivid bright cobalt blue

IMAGE CLUSTER PARTITIONS

Pixels of the image assigned to each cluster. The border is the color of the cluster as calculated by the average value of its pixels.

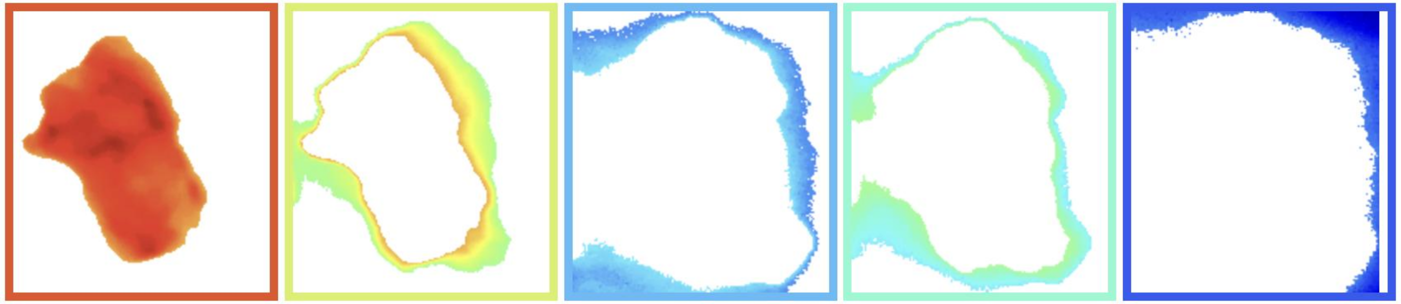

Supplement 1S.G

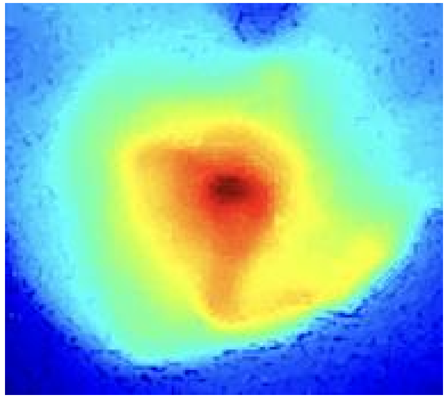

COLOR CLUSTERS

Colors in the image were clustered into **5** groups (k-means). The average color of the colors for each cluster is shown. The name is the closest named color and its distance is shown using ΔE. The tags are the set of words formed by all named neighbours within ΔE ⩽ 5. The list of words above is the set of all unique words in this set of words.

Cluster colors, sized by number of pixels:

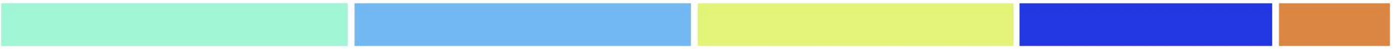

cluster	pixels	name	HEX	RGB	HSV	LCH	Lab	tags
	25.38%	126,244,204<br>light turquoise ΔE=1.9	#83F9D3	131 249 211	160 48 98	90 43 169	90 -42 8	light aqua aquamarine turquoise
	24.68%	101,197,255<br>light brilliant cornflower blue ΔE=5.1	#58BAF9	88 186 249	203 65 98	72 41 256	72 -10 -40	light brilliant cornflower blue
	23.18%	236,255,101<br>light brilliant apple green ΔE=4.2	#DFF561	223 245 97	69 61 96	93 72 112	93 -27 67	light brilliant apple green
	18.60%	0,58,231<br>vivid sapphire blue ΔE=3.6	#1D38EB	29 56 235	232 88 92	36 107 302	36 57 -90	vivid sapphire blue
	8.15%	237,135,45<br>cadmium orange ΔE=3.4	#EA8130	234 129 48	26 80 92	64 68 59	64 35 59	cadmium dusty jaffa side west orange

IMAGE CLUSTER PARTITIONS

Pixels of the image assigned to each cluster. The border is the color of the cluster as calculated by the average value of its pixels.

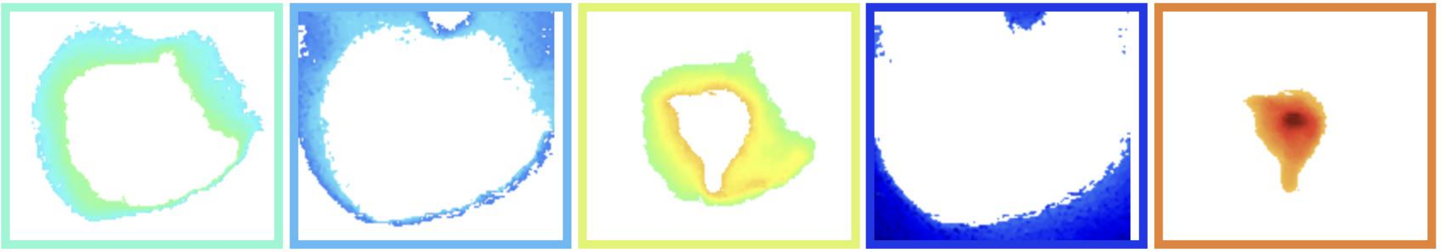

Fluorescence intensity ratio (FIR)

=(8.15%+23.18%)/25.38%

=1.23

Supplement 1S.H

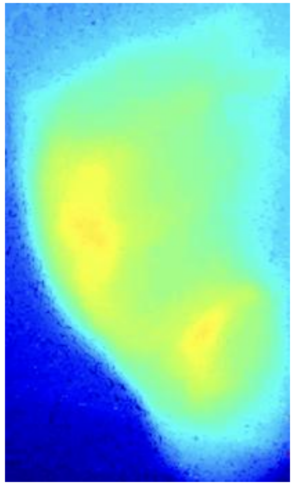

COLOR CLUSTERS

Colors in the image were clustered into **5** groups (k-means). The average color of the colors for each cluster is shown. The name is the closest named color and its distance is shown using ΔE. The tags are the set of words formed by all named neighbours within ΔE ⩽ 5. The list of words above is the set of all unique words in this set of words.

Cluster colors, sized by number of pixels:

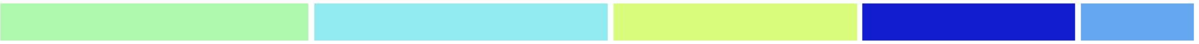

cluster	pixels	name	HEX	RGB	HSV	LCH	Lab	tags
	26.36%	144,253,169<br>foam green ΔE=1.6	#94FCA6	148 252 166	130 41 99	91 57 146	91 -48 32	light very emerald foam green mint
	25.01%	125,249,255<br>electric blue ΔE=3.8	#71EEF2	113 238 242	182 53 95	88 36 200	88 -34 -12	electric blue
	20.76%	203,248,95<br>pear ΔE=2.5	#D1FD67	209 253 103	78 59 99	94 74 118	94 -35 65	light dark brilliant olive pear green lime
	18.23%	1,15,204<br>true blue ΔE=3.7	#0E1DD7	14 29 215	236 94 84	29 110 305	29 63 -91	true blue
	9.63%	101,178,255<br>light brilliant azure ΔE=4.7	#4EA9F8	78 169 248	208 68 97	67 47 267	67 -2 -47	light brilliant azure

IMAGE CLUSTER PARTITIONS

Pixels of the image assigned to each cluster. The border is the color of the cluster as calculated by the average value of its pixels.

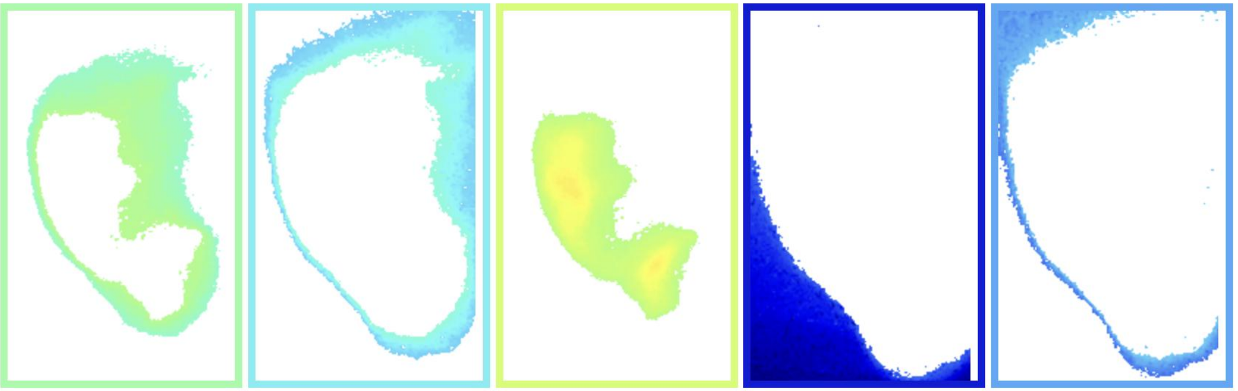

Fluorescence intensity ratio (FIR)

=20.76%/26.36%

=0.79
